# Primary ectopic axillary breast cancer: a case series 

**DOI:** 10.1186/s13256-021-02998-w

**Published:** 2021-07-31

**Authors:** S. Sghaier, M. GHalleb, I. Marghli, A. Bouida, J. Ben Hassouna, R. Chargui, K. Rahal

**Affiliations:** Surgical oncology department, Salah Azaiez Institute of cancer, Tunis, Tunisia

**Keywords:** Ectopic breast tissue, Carcinoma, Surgery, Chemotherapy, Radiotherapy, Hormonotherapy

## Abstract

**Introduction:**

Ectopic breast tissue is present in 2–6% of women. Ectopic breast cancer represents an uncommon disease accounting for about 0.3% of all breast neoplasms, limiting the available evidence. Thus, we aim to report long-term outcomes in five cases treated at our institution.

**Case series:**

Our Tunisian patients’ median age was 48 years (33–60 years), and the median follow-up was 8 years (4–10 years). The ectopic breast tissue was located four times in the right axilla. The median tumor size was 25 mm (15–55 mm). Four of the patients underwent a wide local excision and axillary lymph node dissection. Three of those women had positive lymph nodes; thus, they received adjuvant chemotherapy, radiation therapy, and hormone therapy. The patient with a negative lymph node (case 5) had adjuvant radiation therapy and hormonal therapy. One of the patients (case 1) had a positive supraclavicular lymph node and received radiation therapy, chemotherapy, and hormonal therapy. The latter developed a locoregional relapse after 4 years and was treated with mastectomy and chemotherapy. One patient (case 4) had a distant metastasis after 2 years of follow-up and received chemotherapy. The three other patients were free of relapse during their follow-up period.

**Conclusion:**

Primary axillary breast carcinoma is a rare entity. Despite the paucity of literature, our findings and authors’ recommendations suggest that local excision can be performed safely with promising outcomes in this subset of patients.

## Introduction

Ectopic breast tissue is present in 2–6% of women, usually localized in the axilla [1, 2]. This ectopic tissue may endure similar physiological and benign or malignant pathological variations that affect the normal breast tissue. The axilla represents the most common site, while the sternum area, the infraclavicular region, the epigastrium, and the vulva have also been described [1–3]. Breast cancer is the most common cancer in women, whereas ectopic breast cancer (EBC) represents about 0.3% of all breast malignancies [4, 5]. Like orthotopic breast carcinoma, the ductal subtype is the most frequent type, while the other histological types may also be present [5, 6]. Surgical treatment consists of wide excision of the tumor with lymphadenectomy [4, 5, 7]. Concerning the adjuvant treatment of EBC, it has the same indications as orthotropic breast carcinoma.

We aim to report our experience of long-term outcomes in five cases of EBC treated at our institution and to shed light on this rare occurrence.

## Case series (Table 1: Patients’ characteristics)

**Table 1:** Patients’ characteristics

Patients	Age	Chief complaint	Affected side	Tumor size (mm)	TNM classification	Histologic findings	Surgery	RT	CT
1	60	Nodule	Right	55	T3N3cM0	*ILC, grade II SBR, HR + *Subclavicular node: +	No	Yes	FEC
2	48	Nodule	Left	20	T2N1M0	*IDC, grade III SBR, HR−, HER2 = 3 + *1N+/13 nodes	Wide excision + LND	Yes	FEC
3	53	Nodule	Right	30	T2N1M0	*IDC, grade III SBR, HR−, HER 2 = 2 + *1N+/13 nodes	Wide excision+ LND	Yes	FEC
4	60	Nodule	Right	18	T1N1M0	*IDC, grade II SBR, HR +, HER2 = 1+ *11N+/13 nodes	Wide excision + LND	Yes	FEC
5	33	Nodule	Right	30	T2N0M0	*MC, HR− *9N-/9 nodes	Wide excision + LND	Yes	No

*ILC* invasive lobular carcinoma, *IDC* invasive ductal carcinoma, *MC* medullary carcinoma, *HR* hormonal receptors, *HER2* human epidermal growth factor receptor, *N+ or −* positive or negative lymph node, *RT* radiation therapy, *CT* chemotherapy, *FEC* 5-fluorouracil + epirubicin + cyclophosphamide, *CMF* cyclophosphamide + methotrexate + fluorouracil

We report a case series of five Tunisian patients referred to Salah Azaiez Institute in Tunis for specialized care of EBC.

### Case 1

The patient, a 60-year-old Tunisian woman with no past medical history, presented to our consultation with a right axillary lump evolving for about 6 months.

Physical examination revealed a right axillary firm ill-defined nodule of 55 mm in diameter. We also found firm ipsilateral axillary lymphadenopathies fixed together and suspicious supraclavicular lymph nodes. Breast examination found no apparent anomaly. Breast mammogram was regular.

There was no distant disease on computed tomography (CT) scan.

The patient underwent a core biopsy of the axillary nodule. The histopathological result was concordant with breast carcinoma. It was an invasive lobular carcinoma with a grade II of the Scarff–Bloom–Richardson (SBR) classification and positive hormonal receptors [estrogen receptor (ER), progesterone receptor (PR)]. The supraclavicular node was metastatic. The tumor was staged as T3N3cM0 according to the TNM system.

The decision was to administer six courses of 5-fluorouracil, epirubicin, cyclophosphamide (FEC)-based chemotherapy followed by locoregional radiotherapy (50 Gy are given to the right breast area, the right subclavicular fossa, and the right axilla with a subsequent boost of 12 Gy for the tumor bed) and hormone therapy by tamoxifen. After a satisfactory therapeutic response, we decided to perform a wide excision of the axillary nodule associated with a lymphadenectomy. However, after discussion with the patient, she preferred to remain closely monitored.

After a 4-year follow-up, the patient presented a right breast carcinoma classified as T2N1M0. She underwent a radical mastectomy. Histopathology analysis concluded with a mixed invasive ductal and lobular carcinoma with a grade II of SBR classification that infiltrated the pectoralis major. There were 16 positive axillary lymph nodes out of 24. Then, she had six courses of FEC with no significant side effects.

The patient remained under follow-up and demonstrated no evidence of local recurrence or distant metastasis.

### Case 2

A 48-year-old Tunisian woman with a 15-year past medical history of a right breast cancer. The lesion measured 20 mm in diameter. The patient underwent conservative breast surgery in another department. Histological examination showed an invasive ductal carcinoma, grade I of SBR, and 16 negative lymph nodes. She was then treated by chemotherapy and radiation therapy. The patient was referred to our department for a 2-month left axillary nodule. On physical examination, we found a 20 mm firm nodule with ill-defined boundaries in the left axilla.

**Fig. 1:**
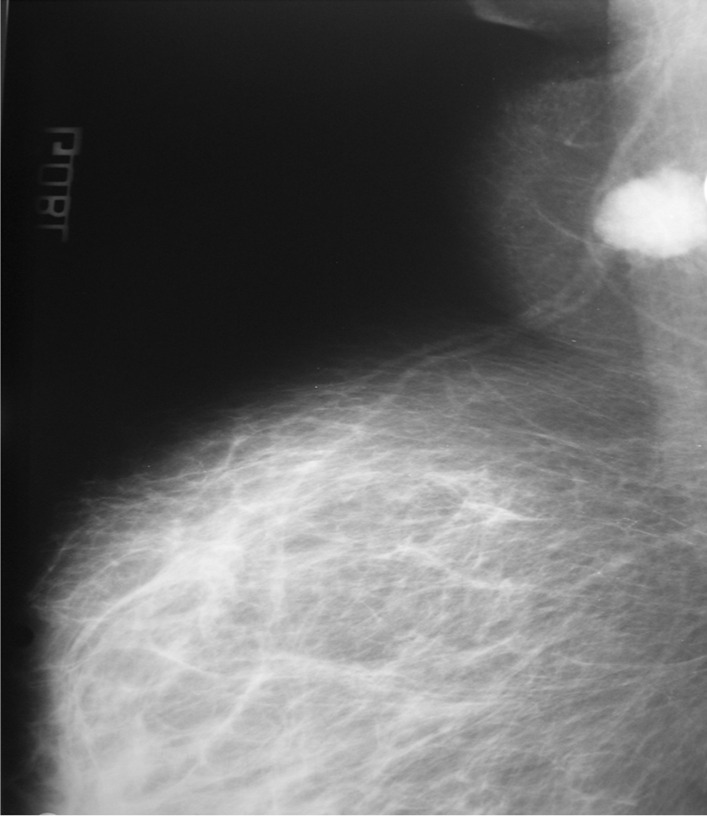
Mammography revealing an ectopic breast ill-defined opacity. A thoracic–abdominal–pelvic scan did not detect any distant metastasis. We staged the tumor as T2N1M0 according to the TNM system

Mammography showed an ectopic breast speculated opacity of 15 mm in size (Fig. 1).

The left axilla mass’s core biopsy was concordant with an invasive ductal carcinoma with an SBR grade III. The patient underwent wide excision of the left ectopic breast tissue and a lymph node dissection. Histopathology confirmed the diagnosis of EBC revealing a 25 mm invasive ductal carcinoma with a grade III of SBR classification, positive HER status (score: 3+), and negative hormonal receptors (ER, PR). There was only 1 metastatic axillary node out of 13.

According to a multidisciplinary meeting decision, the patient received six courses of FEC chemotherapy succeeded by locoregional radiotherapy, including 50 Gy given to the right breast area, the chest wall, and the internal mammary nodes with a subsequent boost of 12 Gy for the tumor bed.

The patient is still under follow-up with no evidence of local or distant relapse.

### Case 3

A 53-year-old Tunisian woman with no past medical history consulted for a right axillary mass developed over the last 4 months. Physical examination revealed a 30-mm firm nodule with unclear limits in the left axilla associated with ipsilateral suspicious lymphadenopathies.

The mammography did not detect any abnormality. The thoracic–abdominal–pelvic scan was free of other distant lesions. The diagnosis of invasive ductal carcinoma was confirmed by the core biopsy of the axilla mass. The tumor was staged as T2N1M0.

The patient underwent wide excision of the right ectopic breast tissue with broad margins and an axillary lymphadenectomy. The histopathologic examination concluded a 22-mm invasive ductal carcinoma, with a grade III of SBR classification, positive HER status (score: 2 +), and negative hormonal receptors.

Then, the patient received six courses of FEC-based chemotherapy. After that, a cycle of radiotherapy was administrated (total dosage of 62 Gy), including 50 Gy to her right breast, the chest wall, and the internal mammary nodes, followed by a boost of 12 Gy for the tumor bed.

After a 2-year follow-up, the patient is in a good state without any evidence of local recurrence or distant metastasis.

### Case 4

The patient, a 60-year-old Tunisian woman with no past medical history, presented with a 12-month history of a right axillary nodule. We found an 18-mm firm nodule in the right axilla on physical examination, which was not well defined. There were suspicious associated axillary lymphadenopathies.

The breast mammogram was regular. A thoracic–abdominal–pelvic scan did not show distant metastasis.

**Fig. 2:**
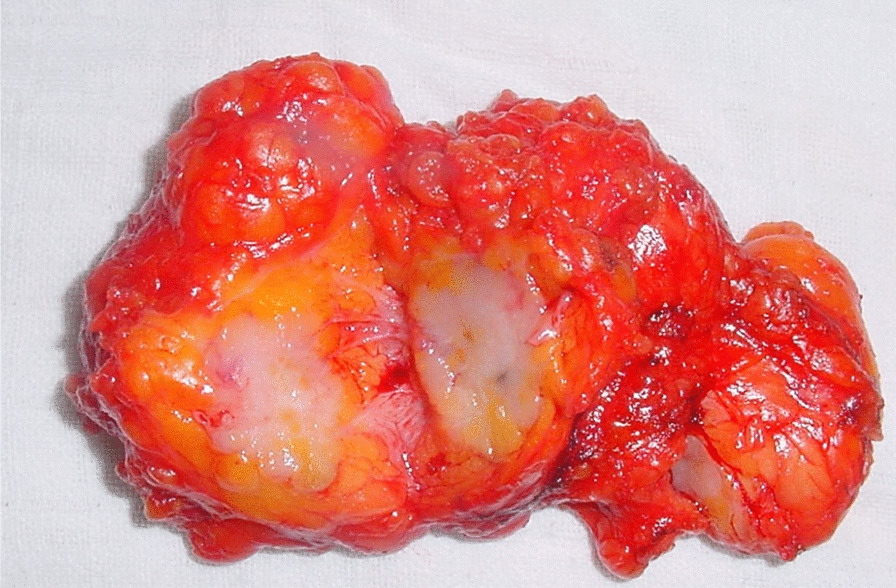
Surgical excision of the ectopic gland containing a tumor nodule of 15 mm

The patient underwent a core biopsy of the axilla nodule, and histopathology showed an invasive ductal carcinoma. The tumor was staged as T1N1M0. The patient underwent wide excision of the ectopic breast nodule and a right axillary lymphadenectomy (Fig. 2).

The histologic type was of invasive ductal carcinoma of 15 mm in size with a grade II of SBR classification and both positive HER status (score: 1 +) and hormonal receptors.

According to the decision of a multidisciplinary meeting, the patient received six FEC chemotherapy courses, followed by radiation treatment with 50 Gy to her right breast, the chest wall, and the internal mammary nodes, succeeded by a boost of 12 Gy for the tumor bed. The patient also received endocrine therapy by tamoxifen.

After a regular follow-up, the patient relapsed with hepatic metastasis that occurred 28 months later.

The patient underwent FEC-based chemotherapy with no significant side effects. After that, she was lost to follow-up.

### Case 5

A 33-year-old Tunisian woman with no past medical or family history consulted for a 1-month right axillary nodule. Physical examination showed a 30-mm firm nodule in the right axilla.

The breast examination showed no abnormalities. There were no associated lymphadenopathies.

A mammogram was performed, revealing an ectopic breast ill-defined opacity.

There were no distant metastases on CT scan. The tumor was staged as T2N0M0.

The patient underwent a right lumpectomy, and the frozen section concluded to the diagnosis of medullary carcinoma. Thus, we conducted a wide resection of the ectopic breast tissue with a lymph node dissection. Histological examination confirmed the diagnosis of medullary carcinoma of 25 mm in diameter with broad safety margins. All the lymph nodes were negative on histologic analysis. There was no expression of hormonal receptors.

The patient received cyclophosphamide, methotrexate, and fluorouracil (CMF)-based chemotherapy associated with radiation treatment with 50 Gy to her right breast, the chest wall, and the internal mammary nodes, followed by a boost of 14 Gy for the tumor bed.

The patient is still in remission after 10 years of follow-up.

## Discussion

There are two types of ectopic breast tissues: supernumerary breast tissue and aberrant mammary tissue [3, 8]. Supernumerary breasts comprise a nipple, an areola, and a mammary gland, whether isolated or associated [1].

Their reported frequency differed among various ethnic groups ranging between 0.6% and 6% [1, 2]. Concerning the aberrant breast tissues, they have neither a nipple nor an areola, and they usually do not involve an organized secretory system [1]. They generally occur close to the breasts [1]. The axilla represents the most common site, while the sternum area, the infraclavicular region, the epigastrium, and the vulva have also been described [1, 3, 9]. Evans *et al*. described that ectopic breast tissue is present in the axilla in 71% of cases [4]. Similarly, Marshall *et al.* mentioned that the axilla was involved in 58% of cases, followed by the sternum region in 18.5%, subclavicular area in 8.6%, submammary area in 8.6%, and the vulva in 4% [1]. They are associated with a frequency that varied between 1.7% and 6% [10]. Like the breast tissue in its anatomical position, the ectopic mammary tissue can undergo physiological changes related to menstrual cycle phases, pregnancy, and even the lactation period [3, 9].

The EBC represents a rare disease, accounting for about 0.3% of all breast carcinomas [4, 5]. It was first described in 1861, and since then, fewer than 200 cases have been reported through literature, affecting mostly the aberrant breast tissue [1]. All of the above publications were isolated cases [11].

A comparison of our results and those reported in the literature are displayed in Table 2. We report in Table 2 the essential studies describing more than one isolated case.

**Table 2:** A comparison of our results and those reported in the literature

Authors	Number of cases	Age	Histology	Treatment	Follow-up
Mornard *et al.* [24]	2	36–62			
Razeman *et al.* [25]	28			4: R Mastect 13: WE+LND	10 recurrences (2 years)
Erdman *et al.* [26]	3				
Khan *et al.* [27]	3			1: R Mastect 2: M Mastect	1: Recurrence-free (9 years) 2: Lost from view
Badejo *et al.* [28]	2			2: R Mastect	2: Recurrence free (2 and 4 years)
Marino *et al.* [29]	2			2: WE + RT	2: Recurrence free (3 years)
Kawahara *et al.* [30]	59	31–84	37 IDC + 3 MC + 1 ApC + 2 Muci C + 1 MC IS	26: WE+LND 26: R Mastect 2: WE	5: Nod. Recurrence 54: Recurrence free (1 month to 13 years)
Haddad *et al.* [31]	2		1 ILC+ 1 U ADK	1: neoadj CT+ WE+LND + RT 1: WE+LND + CT + RT + HT	2 :Recurrence free (1 year)
Yanagi *et al.* [17]	94	26–88	52 IDC+ 5MC+ 2ILC+ 5Muci C+ 4 Ap C	6: neoadj CT + WE + LND or Mastect 84: Surgery (49 WE+ 1 WE+LND+ 3 R Mastect + 18 M Mastect + others) 72: adj treatment (RT +/− CT +/− HT)	9: Recurrences (3 nodal + 2 local + 2 bone + 1 systemic + 1 peritoneal cavity)
Our series	5	33–60	3 IDC + 1 ILC 1 MC	1: CT + RT 4: WE+LND + CT + RT +/−HT	1: Metastasis (2 years) 4: Recurrence free

*ILC* invasive lobular carcinoma, *IDC* invasive ductal carcinoma, *MC* medullary carcinoma, *MuciC* mucinous carcinoma, *Ap C* apocrine carcinoma, *MC IS* medullary carcinoma in situ, *R Mastect* radical mastectomy, *WE+LND* wide excision + lymph node dissection, *M Mastect* modified mastectomy, *neoadj* neoadjuvant, *adj* adjuvant, *RT* radiation therapy, *CT* chemotherapy, *HT* hormone therapy

The primary location reported was the axilla in two-thirds of the cases and the chest wall and vulva in the remaining reports [5]. This disease is difficult to identify preoperatively owing to its scarcity.

It presents mainly as a firm ill-defined mass developed within the ectopic breast tissue. This lesion may be challenging to distinguish from benign axillary masses (lipoma, tuberculosis, reactive lymphadenopathy) or malignant ones (nodal metastasis, adnexal tumors) [5, 12–15].

The imaging findings demonstrated an aspect of the ectopic breast tissue that is identical to that of the breast [16]. Mammography with its oblique and craniocaudal views represents the best incidence angles as the EBC is present in the axilla [16]. However, it may not always be helpful [17]. The EBC appears as an ill-defined speculated opacity within the ectopic breast tissue, independent of the orthotropic breast. Further exploration by ultrasound tests may be beneficial for tumors located at distal sites that are not detected by mammography’s oblique incidence [18].

The diagnosis of EBC is confirmed histologically, demonstrating ductal carcinoma in most cases, whereas all the other histologic subtypes can be present (lobular, medullary, papillary…) [5, 6]. A review of the literature of EBC made in Japan demonstrated that medullary, mucinous, and apocrine carcinoma were more frequently encountered than pectoral breast cancer [17]. In addition to that, Marshall *et al.* noted that the distribution of histological types was as follows: 79% of invasive ductal carcinomas, 9.5% of lobular carcinomas, and 9.5% of medullary carcinomas [1]. In our series, we described one case of invasive medullary carcinoma and another one of lobular carcinoma. Histologic similarities between EBC and benign adnexal tumors increase difficulties in making the right diagnosis [5]. Immunohistochemistry may help attest to hormonal receptors’ status for estrogen and progesterone, which are negative in adnexal tumors [5].

The surgical treatment consisted initially of a mastectomy along with the ectopic breast tissue and lymph node dissection. However, encouraging survival outcomes were described among patients treated only by wide excision of the ectopic gland [4, 5]. Evans *et al.* did not demonstrate any survival advantage for a radical mastectomy when compared with local excision and lymphadenectomy [4]. Cogswell *et al.* discovered that local recurrence can occur with both surgical procedures [10].

Thus, wide surgical excision with broad margins associated with an axillary lymphadenectomy represents the preferred treatment [4, 5, 7]. Nevertheless, it should be noted that no data are comparing both surgical modalities.

Concerning the adjuvant treatment of EBC, it has the same indications as the breast carcinoma in its anatomical position [1, 5, 7, 18, 19].

Some authors consider that the prognosis of EBC is unclear because of the lack of data concerning the follow-up [4, 7]. Others reported that EBC was carrying a prognosis comparable to that of breast carcinoma at the same stage of the disease [18]. However, EBC may spread to regional lymph nodes earlier than breast carcinoma [8, 15, 20, 21]. This could be explained by the fact that the tumor is close to lymph node relays and the diagnostic delay [20]. In the review literature of 94 Japanese patients, lymph node metastasis was found in 51.8% of cases [17]. Correspondingly, Marshall *et al.* reported a rate of 46% of positive axillary lymph nodes [1]. In our series, four patients out of five presented lymph node metastasis despite the tumor’s small size. Visconti *et al.* described that the most affected lymph nodes are homolateral axillary ones and supraclavicular nodes [11].

Due to the scarcity of this disease, we believe that a prophylactic excision may be recommended in patients with risk factors of breast cancer in whom close monitoring is not easy, especially without any functional or aesthetic outcomes [22]. On the other hand, Roorda *et al.* consider that prophylactic excision of all the ectopic breast glands is mandatory as EBC has a poor prognosis [23].

## Conclusion

EBC represents a rare neoplasm, accounting for 0.3% of all breast malignancies. Radiological findings and surgical management of any axillary mass developed within the ectopic breast tissue enable the right diagnosis to be made earlier. Further studies are required to describe this disease’s epidemiologic aspect and give a higher grade of recommendation for its management.

## Data Availability

All the data used were taken from the patient's medical folder available at our institution’s archive.
